# Stereotactic body radiation therapy for multiple lung cancers in a patient with six primary cancers: a case report

**DOI:** 10.1186/s13256-024-04633-w

**Published:** 2024-07-11

**Authors:** Naoko Ishida, Kenji Nagata, Jyunki Fukuda, Yasuo Oguma, Tomonori Hirashima, Kenichi Minami, Yasumasa Nishimura, Yukinori Matsuo

**Affiliations:** 1 Department of Radiation Oncology, Kindai University Faculty of Medicine, 377-2 Onohigashi, Osaka-Sayama, Osaka 589-8511 Japan; 2 Department of Radiation Oncology, Ishikiriseiki Hospital, 18-28 Yayoi-cho, Higashiosaka, Osaka 579-8026 Japan; 3 Department of Radiation Oncology, Kindai University Nara Hospital, 1248-1 Otoda Town, Ikoma, Nara 630-0293 Japan; 4 Radiation Therapy Center, Fuchu Hospital, 1-10-17 Hiko Town, Izumi, Osaka 594-0076 Japan; 5 Department of Thoracic Oncology, Ishikiriseiki Hospital, Higashiosaka, Japan; 6 Department of Respiratory Medicine, Ishikiriseiki Hospital, Higashiosaka, Japan

**Keywords:** Stereotactic body radiation therapy, Multiple primary malignant neoplasms, Multiple lung cancers

## Abstract

**Background:**

Surgery is the standard care for patients with early-stage lung cancer, and stereotactic body radiation therapy is an option for those who are medically inoperable or refuse surgery. Medical developments in diagnostic and therapeutic strategies would prolong prognosis of patients with cancer. The number of patients with multiple cancers has also increased. Duplex primary malignant neoplasms are the most common, and triple or more primary malignant neoplasms were extremely rare. This is the first case of sextuple primary malignant neoplasms with lung cancer.

**Case presentation:**

We report a case of two courses of stereotactic body radiation therapy for an 88-year-old Japanese male patient with six primary cancers in five organs. Cancers were detected in the thyroid, prostate, esophagus, bladder, and lungs. He also had a history of angina pectoris and had undergone percutaneous coronary intervention. Although he was capable of undergoing surgery for lung cancers, he refused it because he had experienced many invasive treatments, such as surgeries and percutaneous coronary intervention. In January 2020, the first stereotactic body radiation therapy was performed for the adenocarcinoma in the right lung. In March 2022, the second stereotactic body radiation therapy was performed for the nodule of the left lung. Although he complained of mild dyspnea after the first stereotactic body radiation therapy, we did not use steroids because his peripheral oxygen saturation was within the normal range. He had pleural effusion, cardiac dilatation, and pericardial effusion 2 months after the second stereotactic body radiation therapy, which improved with the use of compression stockings.

**Conclusion:**

A total of 43 and 17 months have passed since the first and second stereotactic body radiation therapy, respectively, there is no local recurrence and the patient can walk independently. We safely performed stereotactic body radiation therapy twice for our older patient with metachronous early-stage lung cancers. If another new tumor is detected, stereotactic body radiation therapy would be a good treatment option for the functional preservation of organs.

## Background

Surgery is the standard care for patients with early-stage lung cancer, and stereotactic body radiation therapy (SBRT) is a treatment for those who are medically inoperable or refuse surgery [1–3]. It is known that multiple SBRTs for multiple lung cancers are safe and effective treatments [4, 5]. Medical developments in diagnostic and therapeutic strategies would prolong prognosis of patients with cancer. However, the number of patients with multiple cancers has also increased [6]. The prevalence of multiple primary malignant neoplasms (MPMN) reported varies from 0.734% to 11.7% [6]. In the cases of MPMN, duplex primary malignant neoplasms were the most common, and triple or more primary malignant neoplasms were extremely rare [7].

In this report, we describe a case of a patient with six primary cancers in five organs who underwent two courses of SBRT. He had undergone many invasive treatments that he found mentally taxing. SBRT is a good choice for patients such as him because it is painless and the intervention time is relatively short.

## Case presentation

We report a case of an 88-year-old Japanese man with diagnoses of thyroid cancer (pT4N1M0, papillary carcinoma) in 2010, prostate cancer (cT2cN0M0, acinar adenocarcinoma) in 2011, esophageal cancer (T1N0M0, unknown) in 2017, and bladder cancer of ureterovesical junction (pT2N0M0, urothelial carcinoma) in 2019. For thyroid cancer, thyroidectomy and bilateral neck dissection, and 100 mCi of radioactive iodine (RAI) therapy were carried out. When he was diagnosed with prostate cancer, his prostate-specific antigen and Gleason score were 6.34 and 4, respectively. Hormonal therapy was selected after active surveillance. Endoscopic submucosal dissection was carried out for esophageal cancer. For bladder cancer, palliative right lower urethral dissection was carried out. Multiple recurrences in the bladder were detected and he underwent transurethral resection of bladder tumor twice, in 2020 and 2021. No local recurrence or distant metastasis was observed in other tumors. He also had angina pectoris in 2005 and had undergone percutaneous coronary intervention.

He had no family history of cancer. He had smoked two packs of cigarettes per day for 50 years, starting from the age of 20 years.

**Fig. 1:**
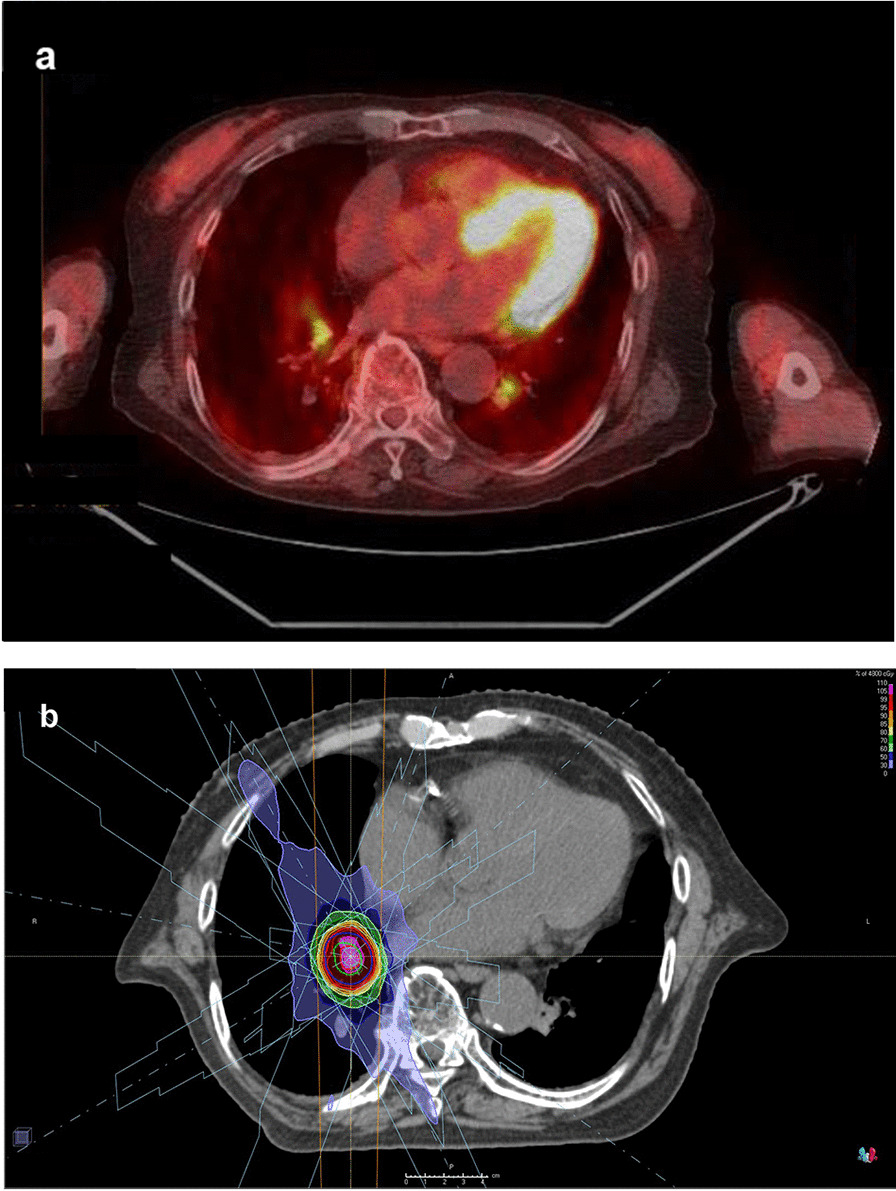
Positron emission tomography and dose distribution. **a** Positron emission tomography before the first stereotactic body radiation therapy.** b** Dose distribution of stereotactic body radiation therapy with 48 Gy

**Fig. 2:**
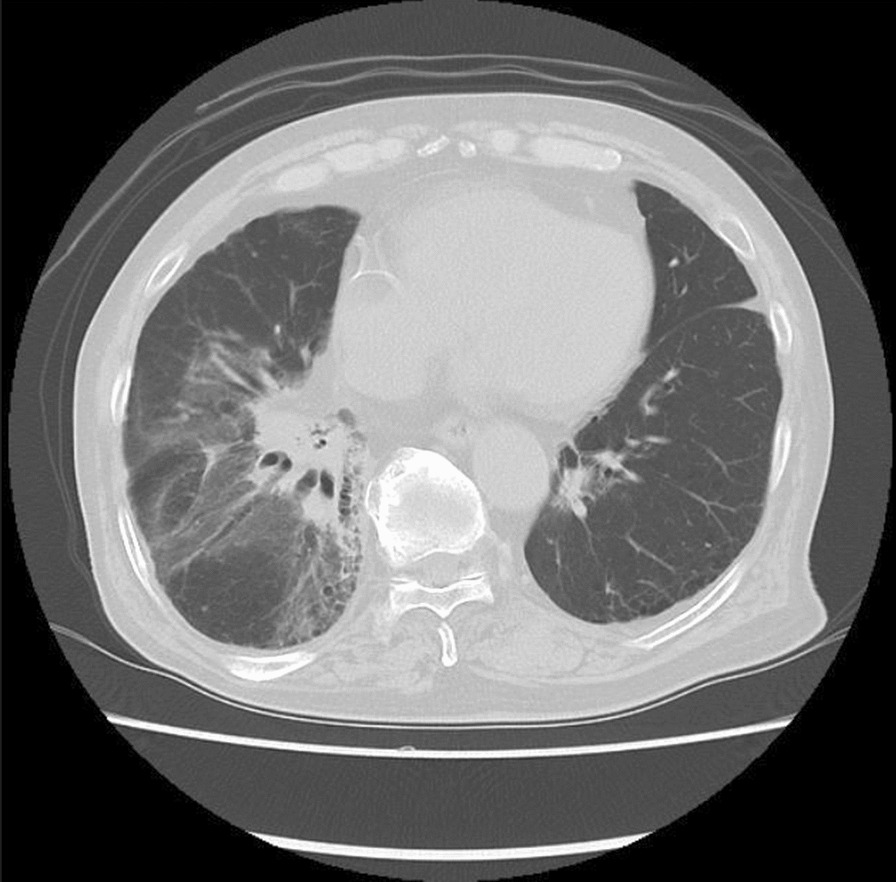
Computed tomography 11 months after the first stereotactic body radiation therapy. Ground-grass appearance consistent with stereotactic body radiation therapy beams was observed

In 2017, computed tomography (CT) scans for screening showed a 7 mm sized nodule in the lower lobe of the right lung. He had no clinical symptoms. It became bigger (15 mm), and positron emission tomography was performed; abnormal activity was found in a nodule in each lower lobe (Fig. 1a). The maximum standardized uptake value (SUV max) of the nodule in right lung lower lobe was 2.9 and in left lung lower lobe was 4.4. Tumor markers carcinoembryonic antigen (CEA) and cytokeratin-19 fragment (CYFRA) were 1.3 ng/ml and 1.6 ng/ml, and they were within normal range. Biopsy by bronchoscopy revealed adenocarcinoma in the nodule in the right lung, and no tumor cells were detected in the specimen from the left lung. The patient’s percent vital capacity (%VC) and percent predicted forced expiratory volume in 1 second (FEV1.0%) were 99.3% and 70.14%, respectively. The patient provided informed consent before SBRT initiation. He refused rebiopsy and operation, and hence, the first SBRT was performed in January 2020 for the right lung cancer, which was staged T1bN0M0 based on the eighth tumor, node, metastasis (TNM) classification of malignant tumors. The prescribed dose was 48 Gy in four fractions with seven beams (Fig. 1b). At 5 months later, the nodule got smaller, but he complained of dyspnea 11 months after the first SBRT. Ground-grass appearance consistent with SBRT beams was observed, which was diagnosed as grade 1 radiation pneumonitis by Common Terminology Criteria for Adverse Events (CTCAE) (Fig. 2) [8]. We did not administer steroids because he had no oxygen desaturation.

The dyspnea became better, and CT scans demonstrated improvement of shadows; however, the nodule in the left lung became larger and it was 2.2 cm in size. We could not observe the nodule in the right lung owing to inflammatory changes. Pulmonary function testing 9 months after the first SBRT revealed a %VC of 94.6% and FEV1.0% of 74.29%.

**Fig. 3:**
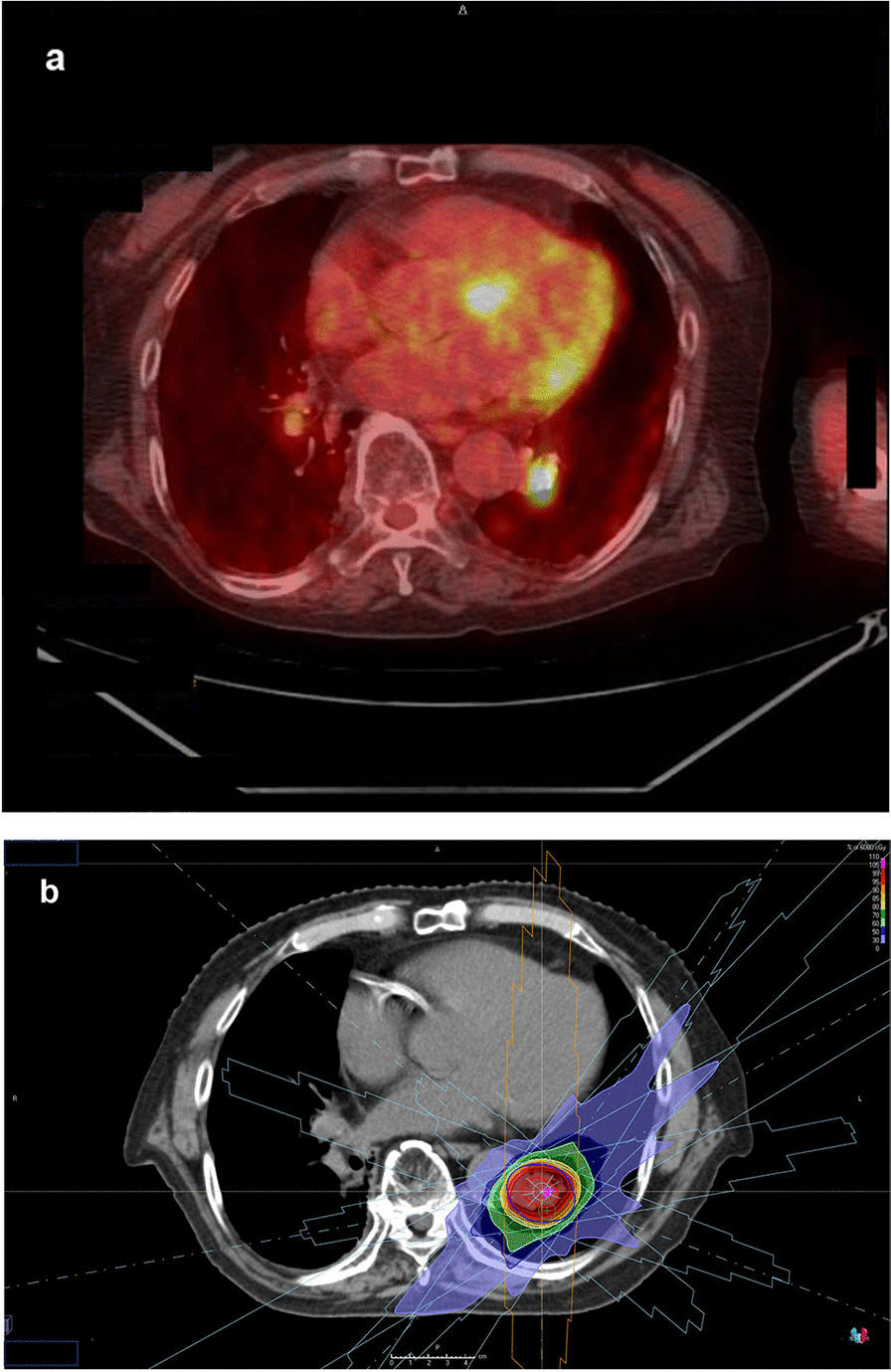
Positron emission tomography and dose distribution. **a** Positron emission tomography before the second stereotactic body radiation therapy. **b** Dose distribution of stereotactic body radiation therapy with 60 Gy

**Fig. 4:**
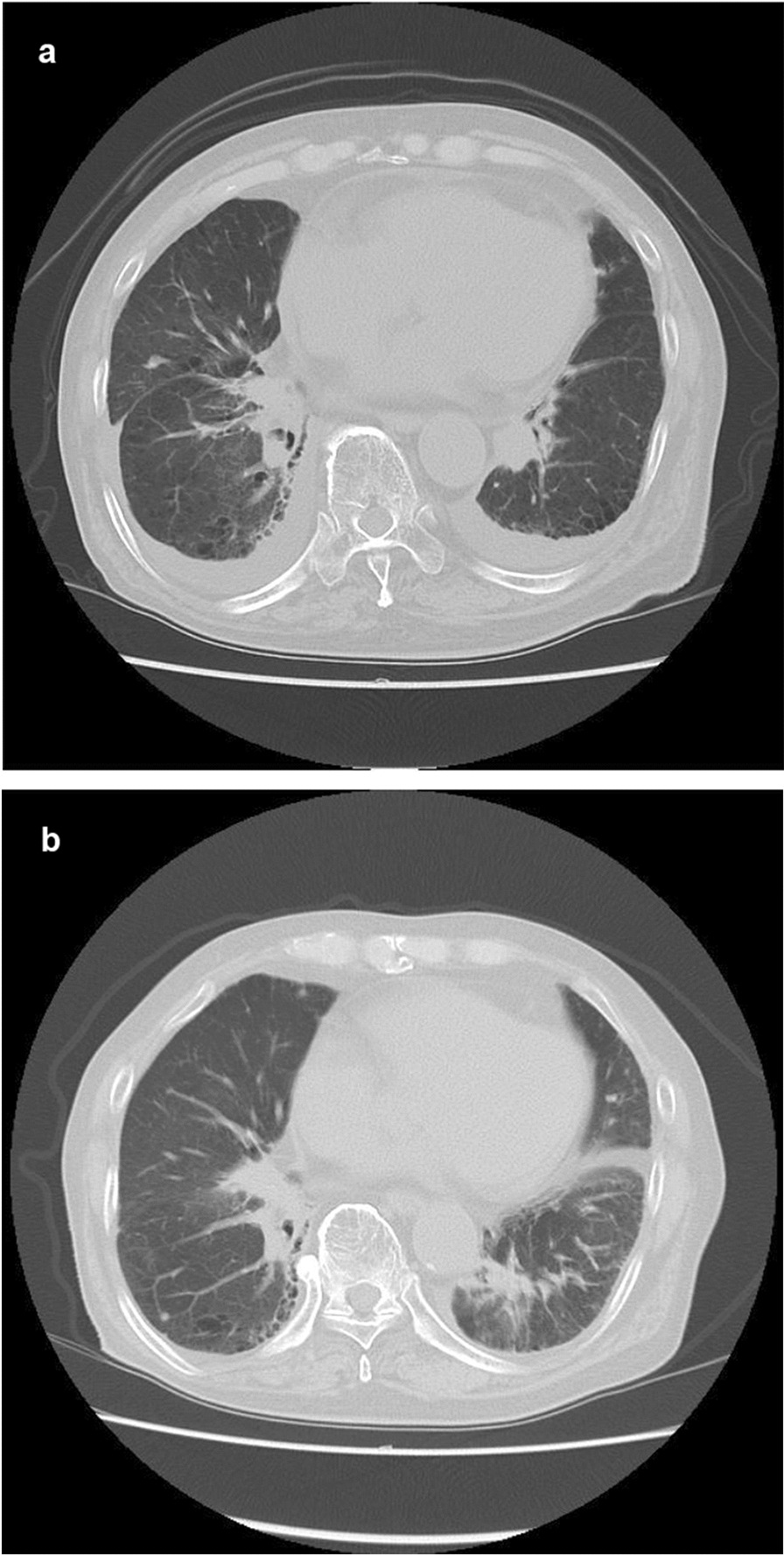
Computed tomography at **a** 2 months after the second stereotactic body radiation therapy and** b** 8 months after the second stereotactic body radiation therapy

Positron emission tomography was performed again, and SUV max of the nodule in left lung lower lobe was elevated to 11.2 (Fig. 3a). We also confirmed no metastasis in the lymph nodes and other organs. CEA was 2.6 ng/ml and within normal range, but CYFRA was elevated to 5.0 ng/ml. We clinically diagnosed the nodule of the left lung as lung cancer, which was staged T2aN0M0 based on the eighth TNM classification of malignant tumors and also as the sixth primary cancer. At that time, the %VC was 92.2% and FEV1.0% was 72.26%. Although the patient was physically fit for surgery, he refused it again. He selected SBRT after receiving informed consent. In March 2022, approximately 2 years after the first SBRT, the second SBRT was performed. The prescribed dose was 60 Gy in eight fractions with six beams (Fig. 3b). At 2 months after the second SBRT, he complained of edema in his legs and palpitations. He had pleural effusion, cardiac dilatation, and pericardial effusion (Fig. 4a). Although right heart strain was detected on the ultrasound cardiogram, left ventricular wall motion was normal, and therefore, he was only advised to use compression stockings. At 8 months after the second SBRT, CT scans demonstrated decreasing pleural and pericardial effusion and shrinkage of the nodule (Fig. 4b). Improvements in his symptoms were also noted. The tumor marker levels were low, and there was no local recurrence.

## Discussion and conclusion

There are some cases with quadruple primary malignancy involving lung cancer in literature [9–11]. However, this is the first case report of six primary cancers involving lung cancer.

When radiation therapy is performed several times, we have to consider summed doses at each organ to prevent severe radiation-related adverse events [12]. Some reports have indicated that multiple SBRTs for multiple lung cancers are safe and effective treatments [4, 5]. However, case reports of several SBRTs being performed in a patient with six primary cancers are rare. In our case, when the two courses of SBRT were performed, the patient was 86 and 88 years old, respectively. Although he was capable of undergoing surgery, he refused it because he had experienced many invasive treatments, such as surgeries and percutaneous coronary intervention.

**Fig. 5:**
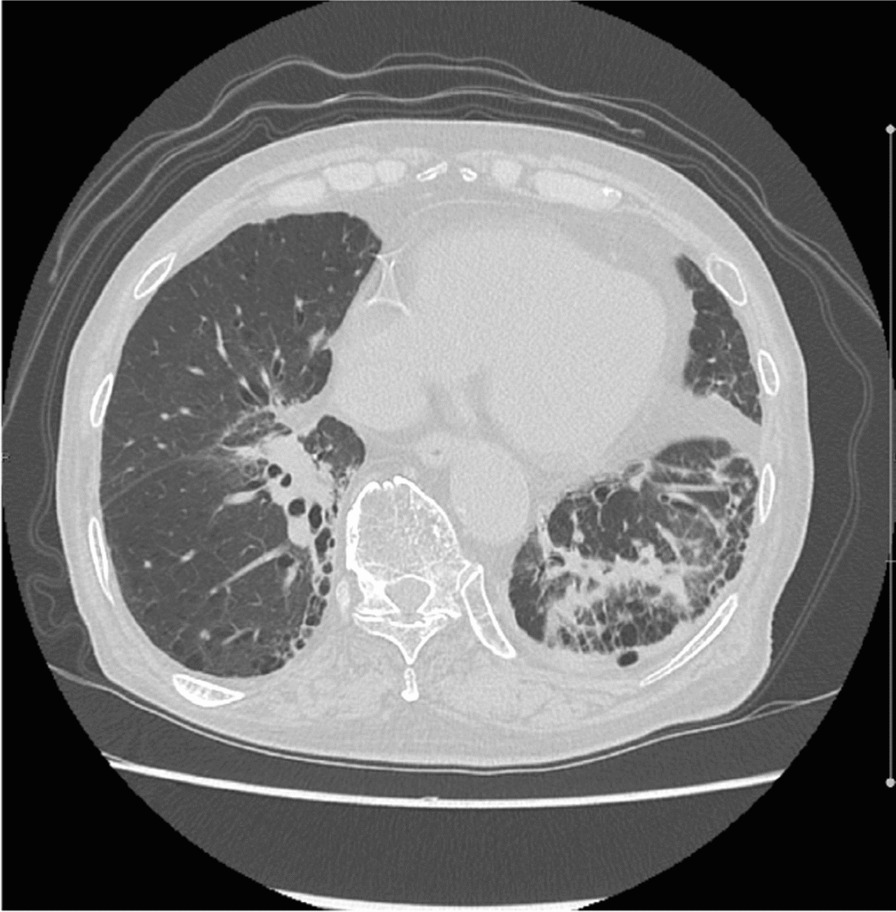
The most recent computed tomography scans. Computed tomography 37 months after the first stereotactic body radiation therapy and 11 months after the second stereotactic body radiation therapy

The most recent CT scans showed shrinkage of the nodule in the left lung, but the nodule in the right lung was not observed owing to inflammatory changes (Fig. 5). Although he complained of mild dyspnea after the first SBRT, his peripheral oxygen saturation was within the normal range. Therefore, we diagnosed grade 1 radiation pneumonitis and did not use steroids [8]. The planning target volume (PTV) of each SBRT was 15.75 cm^3^ and 28.62 cm^3^. We also investigated the equivalent dose in 2‑Gy fractions (EQD2) for α/β of 3of mean lung dose (MLD), V5Gy, and V20Gy (the percentage of lung volume exceeding 5 and 20 Gy) in each SBRT. In fact, MLD was 1.97 Gy EQD2 and 2.56 Gy EQD2, and simple summation was 5.79 Gy. V5Gy of each SBRT was 15.21% and 2.62%, and summation was 29.50%. V20Gy of each SBRT was 2.62% and 5.40%, and summation was 8.04%. We examined %VC and FEV1.0% three times: just before the first SBRT, 9 months after the first SBRT, and just before the second SBRT. Although %VC decreased, pulmonary function was within normal limits. He did not need home oxygen therapy, and we were able to perform SBRTs without declining his quality of life. We have encountered other patients who have safely received SBRT three times. Considering the appearance of another primary cancer or diseases, which needed surgery, SBRT was a good choice to preserve his lungs.

**Fig. 6:**
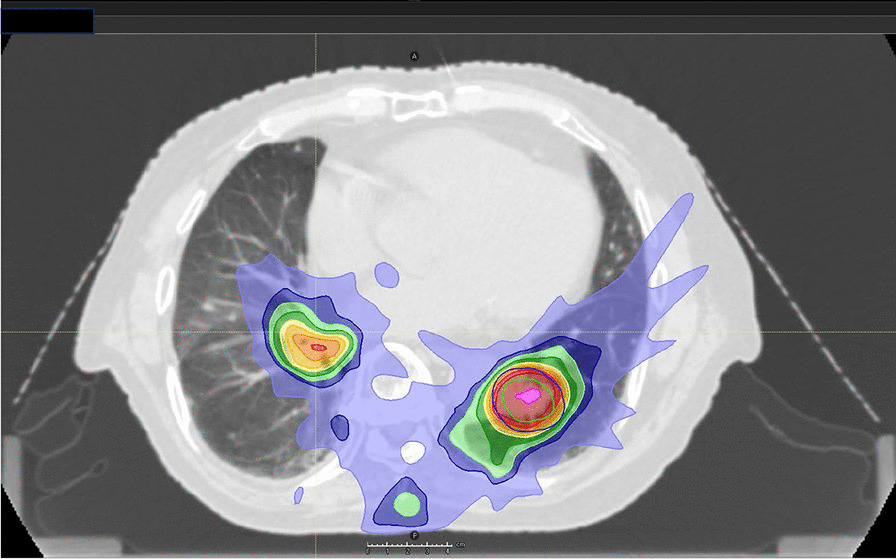
Dose distribution. Dose distribution of dose summation of the first and the second stereotactic body radiation therapy

After the second SBRT, we suspected radiation pericarditis because computed tomography showed pleural and pericardial effusion. Right heart strain was detected on the ultrasound cardiogram and pericardial effusion may have caused it. Both SBRTs used multi-field irradiation and we managed to select beams, which resulted in low cardiac doses (Fig. 6).

Few studies have retrospectively investigated the cardiac dose and its toxicities in early-stage lung cancer SBRT. Reshko *et al*. found that patients with a history of heart problems are at an increased risk of cardiac events [13]. Farrugia *et al*. evaluated the impact of radiation doses to cardiac substructures. They found that the minimum dose administered to 45% (D45%) of the right atrium is correlated with noncancer associated survival and overall survival, and identified a cutoff value of 890.3 cGy using sequential log-rank testing [14]. In this case, the D45% of the right atrium was 725 cGy and 45 cGy in the first and second SBRT, respectively, with a summated dose of 780 cGy. This patient had experienced angina pectoris and may have had an increased risk of developing cardiac events. To prevent radiation pericarditis, we reduced doses per fractionation and increased the number of fractionations. We were able to reduce doses to the right atrium; moreover, we avoided using the beam, which irradiated the heart directly.

A total of 43 and 17 months have passed since the first and second SBRT, respectively, and there is no local recurrence; the patient is still alive and walking independently. For early-stage lung cancer, lung resection and mediastinal lymph node dissection or systematic lymph node sampling were performed. Because SBRT only involves targeted irradiation of the lesion, nodal recurrence might occur.

Although bladder cancer has occurred several times with local recurrence, it has been under local control. The 5-year relative survival rates of thyroid cancer, prostate cancer, and bladder cancer in Japan are 94.7%, 99.1%, and 73.3%, respectively [15], and these cancers have a relatively good prognosis. Longer prognosis would also increase the probability of emerging new cancers. Smoking may be one of the reasons for the six primary tumors in this patient. Moreover, RAI therapy is considered to be a risk factor for second primary cancer (SPC) [16–18]. On the other hand, no relationship between RAI therapy and the risk of SPC was found [19–21]. While most ^131^I is concentrated in the thyroid gland, much smaller amounts are concentrated in salivary glands, stomach, small intestine, bladder, and bone marrow [22]. Ronckers *et al*. found an almost twofold risk among thyroid cancer patients given ^131^I therapy for combined group of cancers in organs that concentrate ^131^I [22]. The patient developed bladder cancer 9 years after receiving internal therapy for thyroid cancer. His bladder cancer may be SPC of RAI therapy. Because he refused to take a genetic test, we could not include genetic abnormalities in this study.

We safely performed SBRT twice for our older patient with metachronous early-stage lung cancers. Several SBRTs being performed in a patient with six primary cancers are rare. SBRT is a good choice for him because it is painless and does not require hospitalization. We will keep examining him regularly for early identification of any potential metastasis, another primary cancer, and severe adverse events of radiation therapy.

## Data Availability

The datasets used during the current study are available from the corresponding author upon reasonable request.

## Abbreviations

SBRT: Stereotactic body radiation therapy
MPMN: Multiple primary malignant neoplasms
RAI: Radioactive iodine
CT: Computed tomography
SUV max: The maximum standardized uptake value
CEA: Carcinoembryonic antigen
CYFRA: Cytokeratin-19 fragment
%VC: Percent vital capacity
FEV1.0%: Forced expiratory volume in 1 second
CTCAE: Common terminology criteria for adverse events
PTV: Planning target volume
EQD2: Equivalent dose in 2‑Gy fractions
MLD: Mean lung dose
V5: The percentage of lung volume exceeding 5 Gy
V20: The percentage of lung volume exceeding 20 Gy in 2‑Gy fractions (EQD2)
D45%: The minimum dose administered to 45% of the structure
SPC: Second primary cancer
